# The efficacy of cytoreductive surgery for oligometastatic prostate cancer: a meta-analysis

**DOI:** 10.1186/s12957-021-02265-8

**Published:** 2021-05-29

**Authors:** Bisheng Cheng, Shuchao Ye, Peiming Bai

**Affiliations:** Zhongshan Hospital Affiliated to Xiamen University, Xiamen, 361001 China

**Keywords:** Prostate cancer, Oligometastatic, Cytoreductive surgery, Endocrine therapy, Meta-analysis

## Abstract

**Backgrounds:**

At present, the application of tumor reduction surgery in oligometastatic prostate cancer has aroused extensive discussion among urologists, but clinicians have not reached a consensus on this issue. The purpose of this study was to evaluate the effect of cytoreductive surgery for patients with oligometastatic prostate cancer by meta-analysis.

**Methods:**

All relevant studies were systematically searched through The Cochrane Library, PubMed, Web of Science, EMBASE, and China Biomedical Literature Database (CBM) up to December 2019. All the previous clinical studies on the comparison of long-term efficacy between the cytoreductive surgery group and the endocrine therapy group were included in the search. The included studies were analyzed using Stata ver.14.0. The research has been registered on PROSPERO website with the registration number of crd42021224316. The relevant registration information can be obtained from the website: https://www.crd.york.ac.uk/prospero.

**Results:**

The case presentation is as follows: ten studies were identified that met the conclusion criteria. The total number of samples was 804; 449 patients underwent cytoreductive surgery, and 355 patients underwent endocrine therapy, and we conducted a meta-analysis of studies to compare the prognosis of endocrine therapy and cytoreductive surgery for treating prostate cancer. After all the studies were analyzed, we found that between cytoreductive surgery and endocrine therapy, a significant difference existed in overall survival (HR = 0.635, 95% CI 0.443–0.908, *P* = 0.013), cancer-specific survival (HR = 0.407, 95% CI 0.243–0.681, *P* = 0.001), and progression-free survival (HR = 0.489, 95% CI 0.315–0.758, *P* = 0.001), while there were no significant difference in progresses to castration-resistant prostate cancer (HR = 0.859, 95% CI 0.475–1.554, *P* = 0.616).

**Conclusion:**

The cytoreductive surgery held advantages in overall survival, cancer-specific survival, and progression-free survival. Therefore, compared with endocrine therapy, cytoreductive surgery could be a more suitable approach in treating oligometastatic prostate cancer.

## Background

The concept of oligometastases was firstly proposed by Professor Hellman and Weichselbaum [1] in 1995. They defined it as a clinical stage between the simple localized state and the extensive metastasis state, that is, the oligometastasis stage. However, there has been enormous controversies about the concept of OM PCA (oligometastatic prostate cancer), and so far, there is no unified treatment for oligometastatic prostate cancer. Experts discussed the topic of oligometastatic prostate cancer at the expert Consensus Meeting on advanced prostate cancer organized by the European Urological Society in 2017 [2]. At the meeting, 61% of the experts identified a limited number of bone and/or lymph nodes as clinically oligometastatic prostate cancer; 10% of the experts believe that oligometastatic prostate cancer is include only limited lymph node metastases; 13% of the experts were more radical, believing that oligometastatic prostate cancer could be limited in number of metastases, including lymph nodes, bone, and viscera. Ten percent of the experts did not believe there was a clinical stage of oligometastasis. Meanwhile, the definition of the number of oligometastases is also controversial. Fourteen percent thought it was less than or equal to 2, 66% claimed that it was less than or equal to 3, and 20% of experts supported less than or equal to 5. Singh et al. [3] analyzed patients with oligometastatic prostate cancer treated with external radiotherapy by retrospective analysis and found that patients with < or = 5 metastatic sites had significantly better survival rates than patients with > 5 lesions. The present accepted definition of oligometastatic prostate cancer is that there are less than or equal to 5 bone metastases, with or without local lymph node metastases, but no visceral metastases [4].

There are still many controversies about the primary treatment of oligometastatic prostate cancer in the world, but the main treatment for oligometastatic prostate cancer is still endocrine therapy. However, there are many evidences that surgical intervention for the primary prostate cancer can improve the prognosis of patients. Tzelepi. V et al. [5] performed endocrine therapy and docetaxel chemotherapy for 1 year in patients with lymph node metastatic prostate cancer and locally advanced prostate cancer After prostatectomy, all specimens were identified by stages according to the recognized standards, and the expression of some molecular markers related to disease progression and treatment resistance was detected by immunohistochemistry and compared with the specimens of 30 untreated patients with high-grade prostate cancer. The results showed that the expression of CYP17, srd5a1, and other molecules in epithelial cells and stroma was increased, and insulin-like growth factor I pathway and other related pathways were activated. More importantly, more than 90% of these tumor specimens still had residual tumor cells with high invasive ability. These results further suggest that some patients without local treatment, including endocrine therapy, cannot completely eliminate the invasive cells in the primary tumor. The mechanism of cytoreductive surgery in the treatment of oligometastatic prostate cancer remains unclear, but its effectiveness has been confirmed by several studies. In other metastatic malignant tumors, cytoreductive surgery has been implemented first, and many successful experiences have been accumulated in metastatic renal cancer, colorectal cancer, lung cancer, and ovarian cancer [6–9]. The main mechanism of tumor reduction surgery is that not only it can reduce the whole body tumor burden, but also reduce the number of tumor cells that fall off from the primary tumor into the blood circulation and decrease the secretion of cytokines that promote tumor growth. Simultaneously, it can improve the sensitivity of follow-up endocrine therapy and chemoradiotherapy [10–13]. In addition, tumor reduction surgery can also alleviate the local symptoms of primary prostate cancer (such as tumor related pain, intractable hematuria, and bladder outlet obstruction caused by prostate cancer), so as to improve the quality of life of patients and improve their ability to tolerate systematic treatment.

Tumor reduction surgery as a treatment for oligometastatic prostate cancer has been confirmed by many retrospective studies. SWOG (Southwest Oncology Group) found that compared with patients who did not receive early tumor reduction radical prostatectomy, 1286 patients with tumor reduction radical prostatectomy had a significantly lower risk of death [14]. The surveillance, epidemiology, and outcome database (the Surveillance, Epidemiology, and End Results database (SEER) is a database of the National Cancer Institute. They retrospectively analyzed 13,692 patients with metastatic prostate cancer (registered between 2004 and 2013). The difference of tumor-specific survival rate between the local treatment group and the non-local treatment group was compared. The results showed that the local treatment group could significantly reduce the tumor-specific mortality rate. In the analysis of the local treatment group, it was found that the prognosis of the tumor reducing radical prostatectomy group was better than that of the radiotherapy group [5]. A retrospective analysis of 1164 patients who underwent radical prostatectomy and extended pelvic lymph node dissection from 2005 to 2012 was conducted in the Cancer Hospital Affiliated to Fudan University in Shanghai, China. Among them, 67 patients with lymph node metastasis after radical prostatectomy and extended pelvic lymph node dissection were followed up for a long time. The results showed that although there was biochemical recurrence, the 5-year tumor-specific survival rate reached 96% [15], and there was significant survival benefit compared with those without local treatment. Another study performed in the same institution also showed that compared with endocrine therapy, transurethral resection of the prostate has certain survival benefits in tumor-specific survival rate and overall survival rate in the treatment of metastatic hormone sensitive prostate cancer [16]. In this study, 39 patients with metastatic hormone sensitive prostate cancer underwent transurethral resection of the prostate, while 107 patients with metastatic hormone sensitive prostate cancer underwent maximum androgen blockade. The results showed that transurethral resection of the prostate can not only improve the symptoms of bladder outlet obstruction, but also reduce serum PSA to a lower level. These results also indicate that patients with metastatic prostate cancer can benefit from survival through tumor reduction surgery.

At present, the application of tumor reduction surgery in oligometastatic prostate cancer has aroused extensive discussion among urologists, but clinicians have not reached a consensus on this issue. Therefore, this study will evaluate the effect of tumor reduction surgery on the prognosis of patients with oligometastatic prostate cancer by means of meta-analysis and further clarify the role of tumor reduction surgery in oligometastatic prostate cancer the efficacy of the treatment of transitional prostate cancer.

## Materials and methods

The work has been reported in line with PRISMA (Preferred Reporting Items for Systematic Reviews and Meta-Analyses) and AMSTAR (Assessing the methodological quality of systematic reviews) Guidelines. The research has been registered on PROSPERO website with the registration number of crd42021224316. The relevant registration information can be obtained from the website: https://www.crd.york.ac.uk/prospero.

### Inclusion and exclusion criteria

The inclusion criteria are as follows: (1) subjects: all patients were confirmed to be prostate cancer by histopathological examination, and tumor metastasis status was determined by CT/MRI/ECT; (2) intervention measures: the experimental group underwent tumor reduction surgery, including radical prostatectomy and transurethral prostatectomy to reduce the number of prostate cancer cells. The control group received endocrine therapy instead of local tumor treatment; (3) the focus of the study was prognostic survival analysis, and sufficient follow-up data were used in this meta-study, that is, the survival rate at each follow-up node could be obtained directly or indirectly from the original text; and (4) type of study: observational or randomized controlled studies. The exclusion criteria are as follows: (1) review and other secondary literature, case reports, conference documents, etc.; (2) animal research; (3) other transition states that do not meet the definition of oligometastases; (4) and literature that fails to obtain the full text of the literature through various methods.

### Outcome measures

The primary outcome was the overall survival rate (OS). The secondary outcomes were tumor-specific survival rate, progression-free survival rate, and progression to castration-resistant prostate cancer.

### Literature screening

Two assessors independently screened the titles and abstracts of the studies. If the relevance of a study was uncertain, the full text was obtained for further evaluation.

### Search strategy

We searched The Cochrane Library, PubMed, Web of Science, EMBASE, and China Biomedical Literature Database (CBM) up to December 2019. We also manual searched the citation lists of the included studies and previous systematic reviews to identify further relevant articles. Taking PubMed as an example, literature retrieval strategies are provided as follows:

#1 (((((((((((((((((“Prostatic Neoplasms”[Mesh]) OR Prostate Neoplasms[Title/Abstract]) OR Neoplasms, Prostate[Title/Abstract]) OR Neoplasm, Prostate[Title/Abstract]) OR Prostate Neoplasm[Title/Abstract]) OR Neoplasms, Prostatic[Title/Abstract]) OR Neoplasm, Prostatic[Title/Abstract]) OR Prostatic Neoplasm[Title/Abstract]) OR Prostate Cancer[Title/Abstract]) OR Cancer, Prostate[Title/Abstract]) OR Cancers, Prostate[Title/Abstract]) OR Prostate Cancers[Title/Abstract]) OR Cancer of the Prostate[Title/Abstract]) OR Prostatic Cancer[Title/Abstract]) OR Cancer, Prostatic[Title/Abstract]) OR Cancers, Prostatic[Title/Abstract]) OR Prostatic Cancers[Title/Abstract]) OR Cancer of Prostate[Title/Abstract]

#2 (((((((((((((((((“Neoplasm Metastasis”[Mesh]) OR Metastases, Neoplasm[Title/Abstract]) OR Neoplasm Metastases[Title/Abstract]) OR Metastasis[Title/Abstract]) OR Metastases[Title/Abstract]) OR metastasis, Neoplasm[Title/Abstract]) OR “Lymphatic Metastasis”[Mesh]) OR Lymphatic Metastases[Title/Abstract]) OR Metastases, Lymphatic[Title/Abstract]) OR Metastasis, Lymphatic[Title/Abstract]) OR lymph node positive[Title/Abstract]) OR lymph node metastasis[Title/Abstract]) OR Bone metastasis[Title/Abstract]) OR skeletal metastases[Title/Abstract]) OR osseous metastasis[Title/Abstract]) OR Bone metastases[Title/Abstract]) OR Oligometastasis[Title/Abstract]) OR oligometastases[Title/Abstract]

#3 (((“Prostatectomy”[Mesh]) OR “Cytoreduction Surgical Procedures”[Mesh]) OR “Transurethral Resection of Prostate”[Mesh]) OR local treatment[Title/Abstract]

#4 #1 AND #2 AND #3

### Data collection

Literature screening is conducted by two researchers independently, and relevant literatures are screened in strict accordance with the previous inclusion criteria and exclusion criteria. Finally, the results are compared one by one. If the results are not consistent, the two researchers can discuss. If the opinions are still inconsistent after discussion, professionals in the field are invited to discuss together. Through intensive reading of the literature, the following data were extracted: first author, year of publication, median age, country, literature type, follow-up time, total number of patients, intervention measures, survival analysis, HR (hazard ratio), 95% CI (confidence interval), median PSA level, Gleason score, TNM stage, and oligometastasis status. The first choice to extract the risk ratio and 95% confidence interval is the data directly given in the paper, and the second choice is the data obtained by Kaplan-Meier curve extraction. The survival rate was extracted by Kaplan-Meier curve, and HR was calculated according to the statistical methods reported in Tierney et al. [17].

The two researchers independently evaluated all the included literature using the NOS scale (Newcastle-Ottawa Scale) [18]. For non-randomized controlled trials, NOS is the most widely used and most reasonable evaluation tool. The content of NOS literature quality evaluation includes three aspects, including 8 sub-evaluation items. The score range of literature evaluation is 0–9. When the score of literature is equal to or greater than 6, we consider it to be of high quality.

### Statistical analysis

The datasets of comparable outcome measures were pooled for meta-analysis using standard statistical procedures in Stata Ver.14.0, and the hazard ratio (HR) was calculated to compare efficacy. Engauge digitizer is a kind of graphics processing software, which is very powerful, and can digitize the information in graphics, and it is free of charge. This software is suitable for research or teaching process; its main function is to extract a variety of images or graphic information, and then digitize them. In this study, “engauge digitizer 4.1” was used to extract the survival rate on the K-M curve. The heterogeneity between studies was evaluated using the *I*^2^ value [19]. When chi-square test *p* ≥ 0.1 and *I*^2^ ≤ 50%, it means that there is homogeneity among the independent studies we included. On this basis, we use fixed effect model to statistically combine the data; when chi-square test *p* < 0.1 or *I*^2^ ≤ 50%, we use fixed effect model to statistically combine the data. When the variance is greater than 50%, it means that there is significant heterogeneity among the independent studies we included. At this time, we need to analyze the source of these heterogeneities (which can be judged by sensitivity analysis and subgroup analysis). If it is judged that these factors have no obvious clinical heterogeneity, we will use the random effect model to merge the data.

## Result

### Study characteristics

A total of 3948 related were screened out. One thousand thirty-six repetitive literatures were excluded by endnote x9; 2861 basic experimental literatures, case reports, reviews, and unrelated literatures related to metastatic prostate cancer or tumor reduction surgery were excluded by reading the title and abstract of the literature (including 396 reviews, 135 conference information, 38 case reports, and 2292 other unrelated literatures). Further assessment of the full text excluded 41 articles that did not conform to the control group inclusion criteria, the number of metastasis was not clear, or the data of prognosis evaluation were incomplete. Finally, 10 literatures [20–29] were included. A total of 804 patients with oligometastatic prostate cancer were included in the 10 articles, including 449 patients in the tumor reduction group and 355 in the endocrine therapy group.

**Fig. 1:**
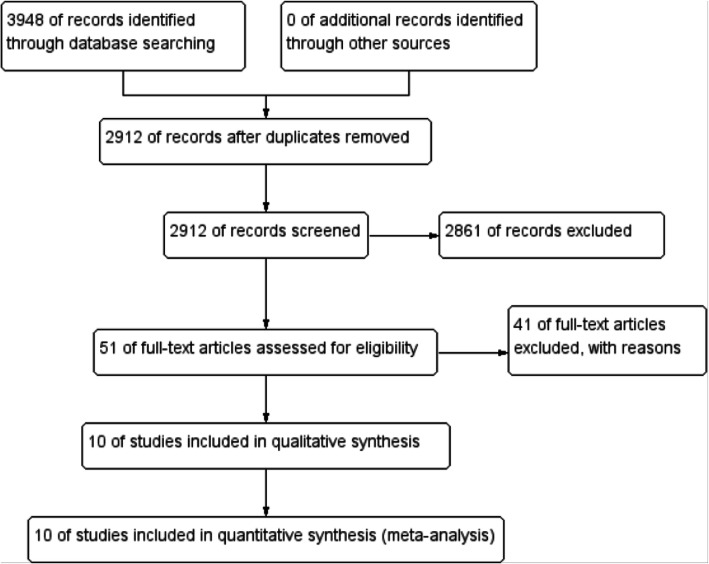
Flow diagram following the PRISMA template of the search strategy

The search process and strategy are shown in Fig. 1. The characteristics of the eligible studies are listed in Table 1. Of the 10 included articles, 4 were included in the overall survival (OS) analysis, 6 in the tumor-specific survival (CSS) analysis (a total of 7 articles were related to CSS indicators, but one of the outcome indicators CSS was excluded because of its short follow-up time), and 4 were used for progression-free survival (PFS) analysis. The experimental group was treated with 6 articles of radical prostatectomy, 1 article of robot-assisted radical prostatectomy, 2 articles of cytoreductive prostatectomy, and 1 article of prostate cancer cryosurgery; the control group (without local treatment) only received endocrine therapy. HR and 95% confidence interval data were directly obtained from three articles [23, 26, 28], and the remaining seven articles [20–22, 24, 25, 27, 29] were obtained by extracting survival rate from Kaplan-Meier curve in the literature, and then converted by EXCEL program designed by Tierney et al. [17].

**Table 1:** The characteristics of included studies for meta-analysis. (CS/ET)

Study	Country	Sample size	Age (median)	Interventions	T-stage	N-stage	M-stage (number of metastases)
Jang et al. [28]	Korea	38/41	65/71	RARP/ADT	T ≤ cT2 5/2 T ≥ cT3 33/39	cN0 21/15 cN1 17/26	≤ 5
Steuber et al. [27]	Germany	43/40	65/70	CRP/ADT	T ≤ cT2 20/9 cT3a/b 23/31	IQR 2–7	= 1 29/16 = 2 9/13 = 3 5/11
Sheng et al. [26]	China	11/13	NA	Cryosurgery/ADT	NA	NA	≤5
Heidenreich et al. [25]	Germany	23/38	61/64	CRP/ADT	T ≤ cT2 7/10 T3a/b 16/24 cT4 0/4	NA	≤5
Steuber et al. [24]	Germany	38/38	65/63	RP/ADT	T1c 8/8 T ≤ cT2 19/19 T ≥ cT3 11/11	Range 1–6	NA
Engel et al. [23]	Germany	132/41	NA	RP/ADT	NA	Range 2–3	0
Grimm et al. [29]	Germany	27/9	64/63	RP/ADT	NA	pN1 27/9	NA
Ghavamian et al. [22]	USA	79/79	65/66	RP/Orchiectomy	T ≤ cT2 42/35 T ≥ cT3 37/44	*n* = 1 16/16 *n* = 2 17/16 *n* ≥ 3 46/47	NA
Schmeller and Lubos [21]	Germany	39/37	62/67	RP/ADT	T ≤ cT2 9 /16 T ≥ cT3 30/21	pN1 27/21 pN2 12/16	0
Cadeddu et al. [20]	USA	19/19	59/59	RP/NA	T ≤ cT2 16/19 T ≥ cT3 3/0	NA	0

*CS* cytoreductive surgery, *ET* endocrine therapy, *NA* no data available, *IQR* interquartile distance, *RP* radical retropubic prostatectomy, *CRP* cytoreductive prostatectomy, *RARP* robot-assisted radical prostatectomy, *ADT* androgen deprivation therapy

### Quality assessment

The 10 articles included in this study were all retrospective control studies. Therefore, the Newcastle-Ottawa scale (NOS) was selected as the evaluation tool. All 10 studies had clear surgical records for the determination of exposure group, and no outcome event occurred before the start of the study. The evaluation of outcome events had a clear definition and data source, and the number of lost visits was within the acceptable range; 6 studies did not mention the source of population in the exposure group or the non-exposure group; 5 studies did not control the confounding factors between the two groups at the same time. The follow-up time was not long enough. There were 3 articles with 8 points in total, 6 articles with 7 points, and 1 article with 6 points. To sum up, we can conclude that all the included literatures are of high quality. The quality evaluation results of the included studies are shown in Table 2.

**Table 2:** Literature quality evaluation table

Year	Selection				Comparability	Outcome		
	1	2	3	4	5	6	7	8
2018	☆	☆	☆	☆	☆	☆	/	☆
2017	☆	/	☆	☆	☆	☆	/	☆
2017	/	☆	☆	☆	☆☆	☆	/	☆
2015	☆	/	☆	☆	☆☆	☆	/	☆
2011	/	☆	☆	☆	☆☆	☆	☆	☆
2010	☆	☆	☆	☆	/	☆	☆	☆
2002	☆	☆	☆	☆	/	☆	☆	☆
1999	/	☆	☆	☆	☆☆	☆	☆	☆
1997	☆	☆	☆	☆	☆	☆	/	☆
1997	/	☆	☆	☆	☆☆	☆	☆	☆

1 Is the case definition adequate?; 2 Representativeness of the cases; 3 Selection of controls; 4 Definition of controls; 5 Comparability of cases and controls on the basis of the design or analysis; 6 Ascertainment of exposure; 7 Same method of ascertainment for cases and controls; 8 Non-response rate

### Efficacy of cytoreductive surgery

#### Overall survival

**Fig. 2:**
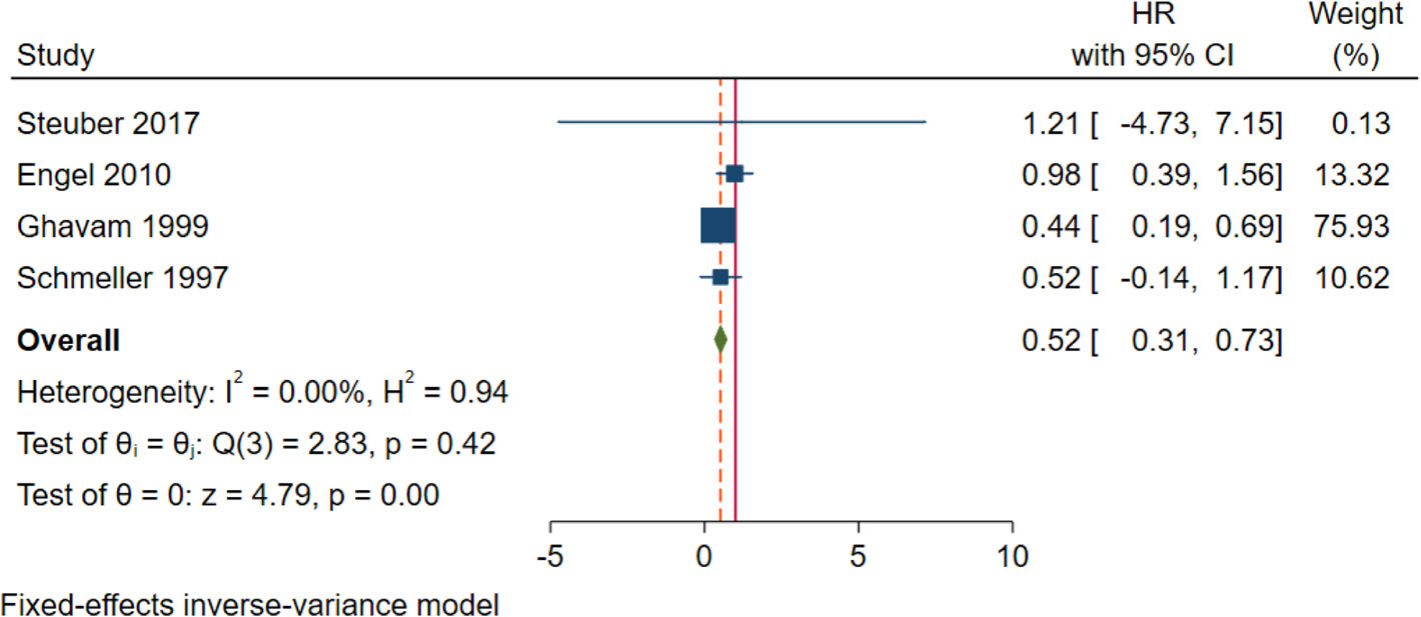
Forest plot of overall survival in tumor reduction group and endocrine treatment group

Ten articles were included, and 4 articles were included to compare the overall survival rate of tumor reduction group and endocrine treatment group. There was no statistical heterogeneity between the results of the included literature (*I*^2^ = 0%, *p* = 0.42). Therefore, the fixed effect model was used for meta-analysis. The results showed that there was a significant difference in OS between the two groups (HR = 0.52; 95% CI 0.31–0.73; *p* = 0.001). It is suggested that tumor reduction can improve the overall survival rate of prostate cancer with few metastases (Fig. 2).

**Fig. 3:**
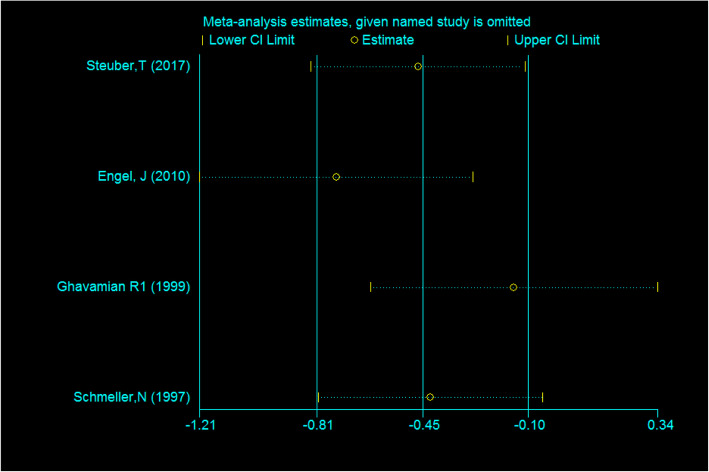
Sensitivity analysis of overall survival indicator

The sensitivity analysis is as follows: each selected study was excluded one by one to observe the impact of deletion on the stability of the overall results. In the overall survival rate group, the results of sensitivity analysis showed that the results of the remaining studies after excluding all the selected studies intersected with the middle equivalent line, which showed that no separate study had significant impact on the outcome, that is, our results were stable, that is to say, the results were reliable (Fig. 3).

**Fig. 4:**
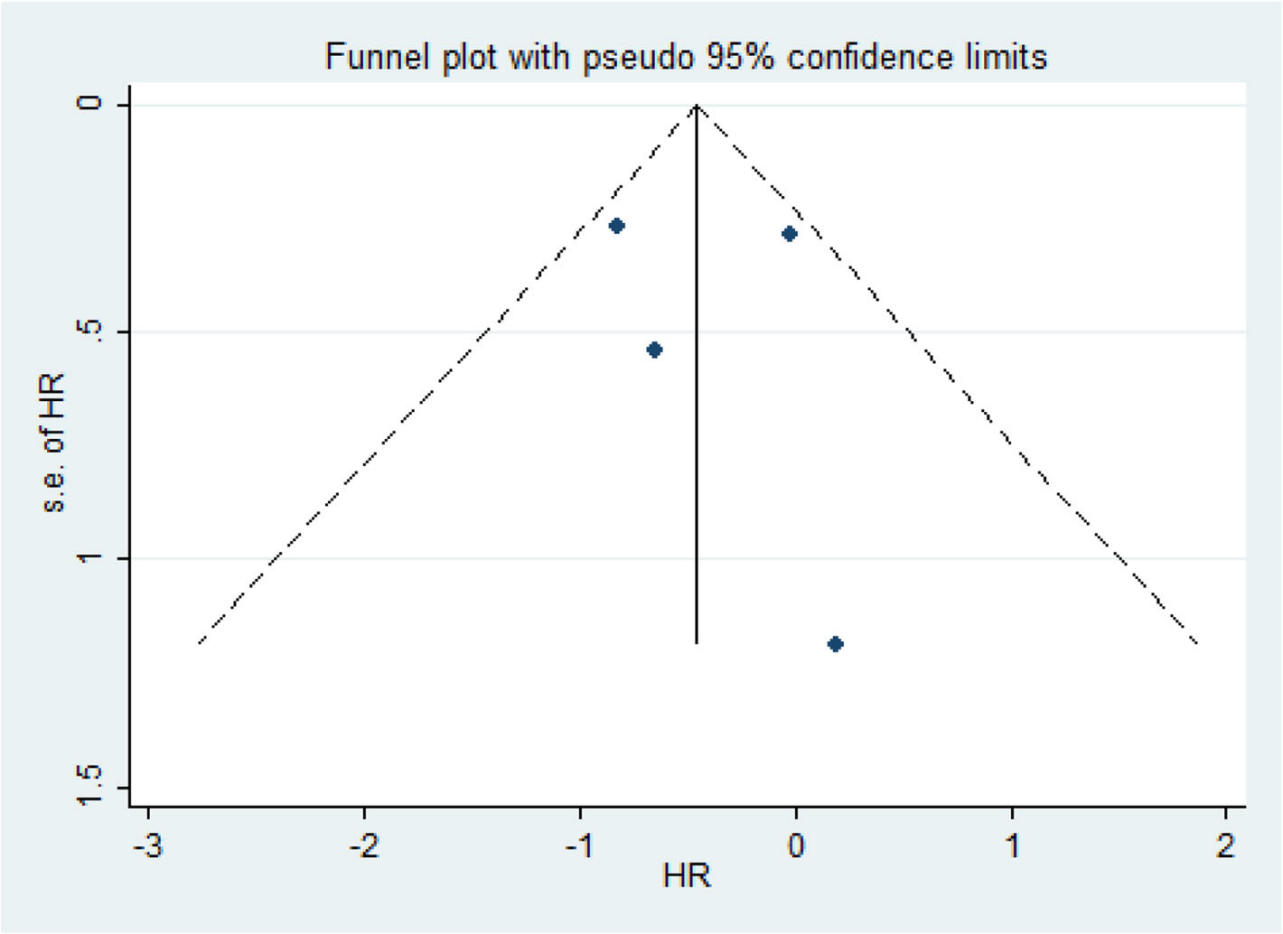
Publication bias assessed with funnel plots of overall survival indicator

Funnel plot and Begg’s linear regression were used to detect publication bias. The funnel plot of overall survival rate showed that both sides of the bisection line were basically symmetrical (Fig. 4); the results of Begg’s linear regression showed that *z* = 0.68, *p* = 0.497, and *p* values were significantly greater than 0.05, which confirmed that there was no publication bias in the included studies.

#### Tumor-specific survival rate

**Fig. 5:**
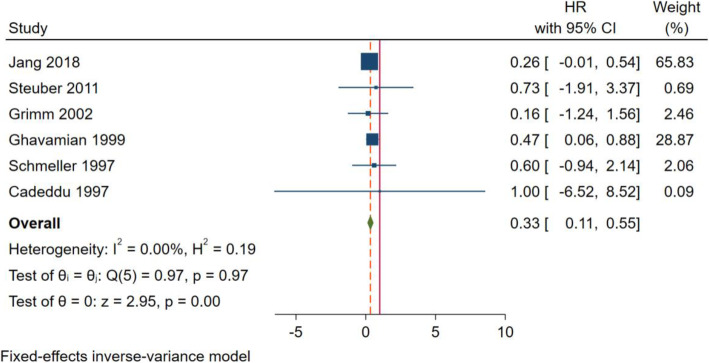
Forest plot of tumor-specific survival in tumor reduction group and endocrine treatment group

Ten articles were included in the study, and a total of 6 articles were included to compare the tumor-specific survival rate between the tumor reduction group and the endocrine treatment group. There was no statistical heterogeneity between the results in the included literature (*I*^2^ = 0%, *p* = 0.97), so fixed effect was used. Meta-analysis showed that there were differences in CSS between the two groups (HR = 0.33; 95% CI 0.11–0.55; *p* = 0.001). It is suggested that tumor reduction can improve the tumor-specific survival rate of prostate cancer with few metastases (Fig. 5).

The sensitivity analysis is as follows: each selected study was excluded one by one to observe the impact of deletion on the stability of the overall results. In the tumor-specific survival group, the results of sensitivity analysis showed that the combined results of the remaining studies after excluding all the selected studies intersected with the median equivalent line, indicating that no single study had a significant impact on the outcome, that is, our results were reliable.

The publication bias test is as follows: funnel plot and Begg’s linear regression were used. The funnel plot of tumor-specific survival rate showed that both sides of the bisection line were basically symmetrical; the results of Begg’s linear regression showed that *z* = 0.56, *p* = 0.573 > 0.05. Therefore, there was no publication bias in the included studies.

#### Progression-free survival and progression to castration-resistant prostate cancer

Ten articles were included, 4 of which were used to compare the progression-free survival rate between the tumor reduction group and the endocrine therapy group. There was no statistical heterogeneity between the results of the included literature (*I*^2^ = 0%, *p* = 0.86). Therefore, the fixed effect model was used for meta-analysis. The results showed that there was significant difference in PFS between tumor reduction group and endocrine treatment group (HR = 0.45; 95% CI 0.22–0.67; *p* = 0.001). It is suggested that tumor reduction can improve the progression-free survival rate of prostate cancer with few metastases. The publication bias test is as follows: no significant publication bias was found.

**Fig. 6:**
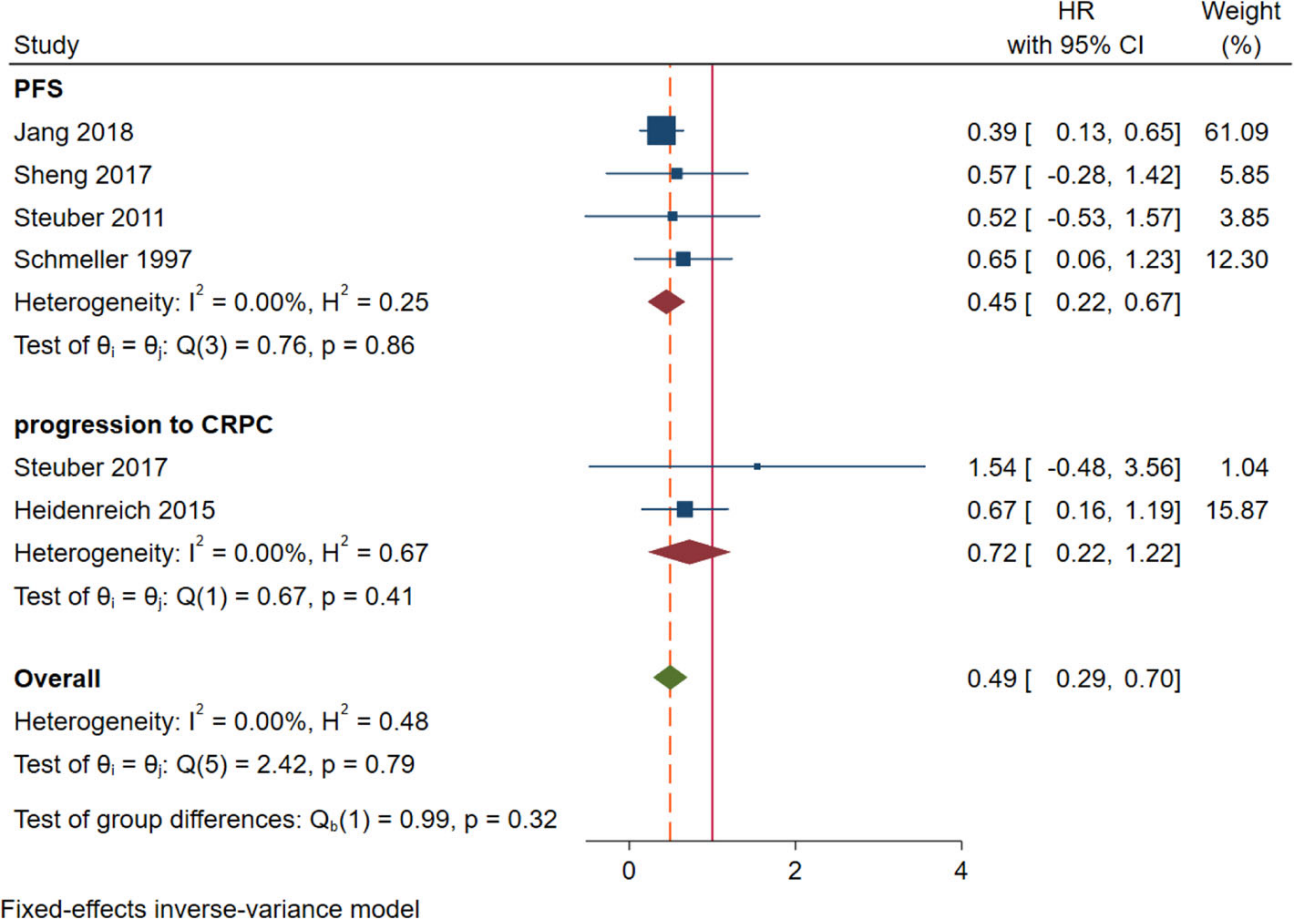
Forest plot of PFS and progression to CRPC indicators

A total of 2 articles compared the progression of castration-resistant prostate cancer between the tumor reduction group and the endocrine therapy group. There was no significant difference in PFS between tumor reduction group and endocrine therapy group (HR = 0.72; 95% CI 0.22–1.22; *p* = 0.41). In view of the small number of included literatures, no further sensitivity analysis and publication bias test were conducted (Fig. 6).

## Discussion

The treatment of metastatic prostate cancer mainly includes endocrine therapy, prostate cancer external radiotherapy, brachytherapy, systemic chemotherapy, and other experimental local treatments. Endocrine therapy and radiochemotherapy, as the main treatment methods of metastatic prostate cancer, have certain curative effect, but they also have many disadvantages, including long treatment cycle and even life-long treatment. There are corresponding side effects such as low libido, erectile dysfunction, gastrointestinal reaction, feminization of male breast, hot flashes, and osteoporosis. In recent years, the concept of oligometastatic prostate cancer has made surgical treatment possible.

Although prostate cancer in this stage is advanced in clinical stage, some studies have found that cytoreductive surgery can improve the long-term survival rate of oligometastatic prostate cancer [30]. Cifuentes et al. [31] performed animal experiments, and they established a mouse model of metastatic prostate cancer. The results showed that the volume of distant metastases in the diseased mice after tumor reduction surgery spontaneously reduced. This experimental result shows that metastatic prostate cancer can benefit from tumor reduction surgery. In addition, many retrospective studies have shown that for patients with metastatic prostate cancer, surgery or radiotherapy can control the symptoms caused by local tumor or metastasis and can make patients with metastatic prostate cancer have survival benefits [32, 33].

Cytoreductive surgery as a part of the treatment options for oligometastatic prostate cancer has become a hot topic in urology, but clinicians have not reached a consensus. Therefore, we used the method of meta-analysis to compare the efficacy of tumor reduction surgery and endocrine therapy in the prognosis of patients with oligometastatic prostate cancer by using prognostic evaluation indicators such as overall survival rate, tumor-specific survival rate, and progression-free survival rate, so as to study the feasibility of tumor reduction surgery in the treatment of oligometastatic prostate cancer.

This is the first meta-analysis on the relationship between tumor reduction and prognosis of oligometastatic prostate cancer. First of all, we searched the major bioinformatics websites at home and abroad according to the limited search terms. Finally, according to the inclusion and exclusion criteria, 10 studies were selected, each of which was a retrospective cohort study. The design was reasonable. The Newcastle-Ottawa scale was used to evaluate the quality of literature. The scores of each study were more than 6. Therefore, this study has high credibility. To sum up, the results of this meta-analysis show that compared with endocrine therapy, tumor reduction surgery can improve the overall survival rate, tumor-specific survival rate, and progression-free survival rate of patients with oligometastatic prostate cancer, suggesting that tumor reduction surgery may be a better method for the treatment of oligometastatic prostate cancer.

There are limitations in this study: firstly, although the total number of patients included is large, there are few literatures included for each prognostic analysis index. Among them, there are only 4 articles in the overall survival rate group, 6 articles in the tumor-specific survival rate group, 4 articles in the progression-free survival rate group, and only 2 articles in the castration-resistant prostate cancer group. This makes it difficult to further subgroup analysis of the results. Secondly, in the included studies, only local lymph node metastasis was found in 6 studies, but no bone metastasis was found. In the other four studies, bone metastasis combined with local lymph node metastasis was found. Therefore, their clinical stages were different, which made the overall clinical stage of all the included patients different, and two large sample studies used for OS analysis did not provide information of metastasis, which may have potential risk of bias. We look forward to the publication of more high-quality research data. Third, the main source of patients included in the study is patients from Europe and the USA, while patients from China are less, which makes the guiding value of this study for domestic patients with oligometastatic prostate cancer reduced. Fourth, all the included studies are retrospective cohort studies, and the level of evidence is lower than that of randomized controlled trials. There may be selection bias, reporting bias, and follow-up bias in the research process, which also reduces the reliability of the conclusions of this study to a certain extent. Fifthly, the retrieval literature span is large, the earliest literature is 1997, the latest literature is 2018, the research span is more than 20 years, and the earlier research may not represent the current diagnosis and treatment behavior, including diagnostic technology, surgical skills, follow-up observation indicators, and other aspects, which may be the main source of bias in the results of this meta-analysis.

## Conclusion

The cytoreductive surgery held advantages in overall survival, cancer-specific survival, and progression-free survival. Therefore, compared with endocrine therapy, cytoreductive surgery could be a more suitable approach in treating oligometastatic prostate cancer. More random clinical trials that are large-sample and followed up long are needed in the future to assess these two approaches.

## Data Availability

The material of this article is original research. All data in this manuscript are available and transparent for readers.
